# Optimizing Cardiac Out-Put to Increase Cerebral Penumbral Perfusion in Large Middle Cerebral Artery Ischemic Lesion—OPTIMAL Study

**DOI:** 10.3389/fneur.2017.00402

**Published:** 2017-08-10

**Authors:** Hannah Fuhrer, Albrecht Günther, Jan Zinke, Wolf-Dirk Niesen

**Affiliations:** ^1^Department of Neurology, Albert Ludwig University of Freiburg, Freiburg, Germany; ^2^Department of Neurology, Friedrich Schiller University Jena, Jena, Germany

**Keywords:** acute stroke therapy, hemodynamics, cardiac output, cerebral perfusion, functional outcome, cerebral infarction

## Abstract

**Introduction:**

In unsuccessful vessel recanalization, clinical outcome of acute stroke patients depends on early improvement of penumbral perfusion. So far, mean arterial blood pressure (MAP) is the target hemodynamic parameter. However, the correlations of MAP to cardiac output (CO) and cerebral perfusion are volume state dependent. In severe subarachnoid hemorrhage, optimizing CO leads to a reduction of delayed ischemic neurological deficits and improvement of clinical outcome. This study aims to investigate the effect of standard versus advanced cardiac monitoring with optimization of CO on the clinical outcome in patients with large ischemic stroke.

**Methods and analysis:**

The OPTIMAL study is a prospective, multicenter, open, into two arms (1:1) randomized, controlled trial. *Sample size estimate*: sample sizes of 150 for each treatment group (300 in total) ensure an 80% power to detect a difference of 16% of a dichotomized level of functional clinical outcome at 3 months at a significance level of 0.05. *Study outcomes*: the primary endpoint is the functional outcome at 3 months. The secondary endpoints include functional outcome at 6 months follow-up, and complications related to hemodynamic monitoring and therapies.

**Discussion:**

The results of this trial will provide data on the safety and efficacy of advanced hemodynamic monitoring on clinical outcome.

**Ethics and dissemination:**

The trial was approved by the leading ethics committee of Freiburg University, Germany (438/14, 2015) and the local ethics committees of the participating centers. The study is performed in accordance with the Declaration of Helsinki and the guidelines of Good Clinical Practice. It is registered in the German Clinical Trial register (DRKS; DRKS00007805). Dissemination will include submission to peer-reviewed professional journals and presentation at congresses. Hemodynamic monitoring may be altered in a specific stroke patient cohort if the study shows that advanced monitoring is safe and improves the functional outcome.

## Introduction

Acute therapy in stroke focuses on restoring and optimizing the cerebral perfusion (CP) to reduce the final infarction volume and improve the clinical outcome (1, 2). Guidelines advocate early recanalization measures by intravenous thrombolysis and/or thrombectomy as a standard of care. In insufficient and unsuccessful vessel recanalization, clinical outcome of acute stroke patients depends on early improvement of penumbral perfusion. The individual evolution of good collateral vessels is a crucial factor for the development and maintainability of penumbral perfusion (1). So far, mean arterial blood pressure (MAP) is the standard target parameter for improving CP being a readily accessible parameter on one hand and expected to be related to CP due to vascular autoregulation on the other (3–5). It has been shown that an increase of MAP levels can reduced the infarction volume and better functional outcome due to improved penumbral perfusion (6). Physically, MAP itself results from a product of cardiac output (CO) and systemic vascular resistance (SVR; 6).

However, former studies have shown that correlation of MAP, and organ perfusion depends on volume state (7, 8), whereas CO has a linear correlation with perfusion (5, 9). Especially in the critically ill, optimized CO correlated with increased organ oxygen delivery, and thus leads to improvement of clinical outcome (10).

In patients with severe subarachnoid hemorrhage (SAH) increased CO during constant, MAP levels lead to a higher cerebral blood flow reducing the rate of delayed cerebral ischemia (11, 12) and improving the functional outcome (13).

The Optimizing cardiac out-Put To Increase cerebral penumbral perfusion in large Middle cerebral Artery ischemic Lesion (OPTIMAL) study aims to investigate the effect of advanced cardiac monitoring with optimization of CO levels in stroke patients. We hypothesize that advanced monitoring reduces the infarction volume and improves clinical outcome.

## Methods and Analysis

### Design

The OPTIMAL study is a prospective, multicenter, open, two-armed, randomized, and outcome-blinded trial. The participating centers are part of the IGNITE network.

### Patient Population—Inclusion and Exclusion Criteria

All subjects with an ischemic stroke are eligible for participation if the following inclusion criteria are met:

– Ischemic stroke ≥30% of the territory of the middle cerebral artery (visual assessment of CT or MR scans).
– Age between 18 and 85 years.
– Within 12 h of symptom onset/12 h from time last seen well.
– Patients without recanalization measures: inclusion within 3 h after hospital admission.
– Patients with recanalization therapy (i.v. thrombolysis, thrombectomy, or both): inclusion within 3 h after cessation of recanalization measures and persistent neurological deficit [with National Institute of Health Stroke Scale (NIH-SS) >10 points]. If patients are in a sedated state and cannot be clinically monitored, the vessel occlusion must be persistent (depicted by angiography or sonography).
– Written informed consent by the subject herself/himself, her/his legal representative or written/oral consent by a close relative or friend according to the patient’s presumed will and interest of participating in this study.

Subjects will be excluded from the trial if any of the following criteria is fulfilled:

– Patients receiving recanalization measures: patients presenting with NIH-SS ≤10 points during recanalization measures or within 3 h after cessation of recanalization measures.
– Intracerebral hemorrhage.
– Premorbid modified Rankin (mRS) score ≥2.
– Severe comorbidities or malignant disease leading to a relevant impact on the 3 months outcome.

### Randomization

Subjects are randomly assigned to standard monitoring or standard plus advanced monitoring using an online-randomization tool (www.randomizer.at). Randomization allocates patients 1:1 to one of the treatment arms; randomization factors are age, gender, hemisphere, NIH-SS, and recanalization measurements.

### Treatment or Intervention

All participating centers have adequate experience in the intensive care management of acute ischemic stroke.

#### Control Arm: Standard Monitoring and Therapy

All patients receive best medical care according to the standards based on the recommendations of the German stroke association. MAP levels of ≥80 mmHg have to be achieved for 48 h; volume (crystalloids) or catecholamines (noradrenaline and dobutamine) are to be administered if needed. According to our hospital standard of care, systolic blood pressure levels are limited to 160 mmHg in patients who underwent recanalization measures.

#### Treatment Arm: Standard Plus Advanced Monitoring and Therapy

Patients receive standard plus advanced cardiac monitoring. Those monitoring systems (“Vigileo,” “PiCCO”) perform an analysis of the arterial pulse contour and calculate a cardiac index (CI; index results from CO and stroke volume related to body surface). Optimizing both parameters with MAP levels ≥80 mmHg and CI ranging between ≥3.0 and ≤4.5 has to be achieved for 48 h with volume (crystalloids) or catecholamines (noradrenaline and dobutamine) administration if necessary. According to our hospital standard of care, systolic blood pressure levels are limited to 160 mmHg in patients who underwent recanalization measures.

The monitoring systems calculate further parameters, which can be used to guide hemodynamic therapy: as a preload indicator the stroke volume variety (SVV) is calculated by (SVmax − SVmin)/SVmean with SVV >10% being a sign of volume depletion. The SVR index indicates vessel dilation and/or volume depletion [normal SVRI levels 1,700–2,400 dyn s/cm^5^/m^2^ (13)]. So, in patients with CI <3.0, crystalloids should be administered as a first step of hemodynamic treatment until SVV <10%. If this measure does not increase the CI >3, noradrenaline, a peripheral vasoconstrictor, according to SVR levels should be used. Dobutamine, as a positive inotropic substance, can be used as last option.

### Primary Outcomes

The primary outcome is a dichotomized mRS score after 3 months score 0–3 versus 4–6 is compared within the treatment groups.

### Secondary Outcomes

Dichotomized outcome of mRS score 0–3 versus 4–6 after 6 months, as well as 0–2 versus 3–6 after 3 and 6 months, change of NIH-SS on day 1, 2, 3, and 10 ± 3 are analyzed. Final infarction volume is calculated. Monitoring parameters (CI and MAP levels) as well as amount of fluid administration and catecholamines are analyzed. Furthermore, safety assessments include the following: in hospital mortality, days of hospital stay, number of secondary intracerebral hemorrhages, and duration and quantity of catecholamine treatment.

### Data Monitoring Body

Data will be recorded in every participating center, and all data will be finally analyzed in the principle center (University Freiburg). Safety parameters will be thoroughly assessed (c.f. secondary outcomes).

### Sample Size Estimates

Sample size calculations are based on the study of Mutoh et al. (13). Sample sizes of 150 for each treatment group (300 in total) ensure an 80% power to detect a difference of 16% in proportions of patients with mRS scores of 0–3 at 3 months at a significance level of 0.05.

### Statistical Analyses

Statistical analysis will be performed as intention to treat. Summary statistics will be used for descriptive analysis. *P*-values for group comparisons and corresponding 95% confidence intervals will be calculated using two-sample *t*-testing, the Mann–Whitney *U*-test, or χ^2^-testing as applicable.

### Study Organization and Funding

The steering committee consists of the project leader Wolf-Dirk Niesen and the principle investigators of the participating centers. The study received funding for the patient randomization by the German Society for Neurointensive Care and Emergency Medicine (DGNI). Besides this, the study is exclusively driven by internal means of the participating centers.

### Stepwise Procedures

Patients with acute ischemic stroke are screened for in- and exclusion criteria when admitted to the hospital. Randomization takes place within 3 h when no acute recanalization measures take place or within 3 h after cessation of recanalization measures. From then on, monitoring and optimizing of hemodynamic parameters are applied for the next 48 h in both groups. Data of NIH-SS scores are collected on day 1, 2, 3, and 10 ± 3, the data of mRS scores at time of discharge and after 3 and 6 months. A follow-up MRI is performed on day 10 ± 3.

### Anticipated Results

The OPTIMAL study aims to investigate the effect of advanced hemodynamic monitoring with optimization of CO levels in stroke patients. We anticipate an advanced monitoring to improve clinical long-term outcome and reduce the infarction volume.

## Discussion

The penumbra concept describes tissue at risk due to hypoxemia around the infarction core. The fate of the penumbral tissue seems to be individually variable and depends on slight differences in perfusion (14). Cerebral collaterals are dynamically recruited after arterial occlusion. Patients being able to develop good collateral flow and therefore restore the penumbra longer seem to develop smaller infarctions. Furthermore, they seem to benefit most from perfusion strategies (1). Based on this penumbra concept, the timeframe of a maximum of 12 h after symptom onset until inclusion in this study was adopted (2).

Patients being treated with an i.v. thrombolysis and/or thrombectomy are excluded from this study within the first 3 h after completion of recanalization therapy due to the increased risk of secondary hemorrhages (15). The blood–brain barrier is abnormally permeable, and treatment measures in the very early acute phase may precipitate or enhance hemorrhagic transformation (16). Therefore, patients can be included into this trial 3 h after the cessation of recanalization measures.

With focus on outcome parameters and since there are no hemodynamic data on severely ill stroke patients, we based our study on data of Ref. (13). They were able to show that advanced hemodynamic monitoring can reduce the incidence of DCI and therefore patients with severe SAH benefited from advanced monitoring with a significant better outcome with a difference concerning independency (mRS 0–3) of 16% (13). Based on results of former studies that standard monitoring poorly reflects actual circulating blood volume and organ perfusion but fluid status effects brain perfusion (5, 7, 8, 13), we hypothesize that advanced hemodynamic monitoring can reduce the final infarction volume in large ischemic stroke. Thus, we adopted the dichotomized primary endpoint of mRS score at 3 months aiming to show a positive effect of advanced hemodynamic monitoring with a shift to a more favorable outcome.

We believe that results of OPTIMAL will provide reliable data on hemodynamic monitoring and functional outcome in large ischemic stroke, thereby forming the basis of subsequent future studies.

## Ethics Statement

The trial was approved by the leading ethics committee of Freiburg University, Germany (Reference Number: 438/14, date of approval: 29.01.2015) and the local ethics committees of the participating centers. The study is performed in accordance with the Declaration of Helsinki and its subsequent amendments and the guidelines of Good Clinical Practice. The study is registered in the German Clinical Trial register (DRKS; DRKS00007805). Dissemination will include submission of study results to peer-reviewed professional journals and presentation at congresses. Hemodynamic monitoring may be altered in stroke patients or in a specific stroke patient cohort if the study shows that advanced monitoring is safe and improves the functional outcome.

## Author Contributions

HF has performed literature research and put the topic in perspective of current knowledge. HF and W-DN have created the study design. AG and JZ gave support of creating the study design. Writing of the article has been performed by AG and JZ, as well of HF and W-DN.

## Conflict of Interest Statement

The authors declare that the research was conducted in the absence of any commercial or financial relationships that could be construed as a potential conflict of interest.
